# Evaluation of cryptococcal antigen testing using a novel chemiluminescence assay in two medical centers of China

**DOI:** 10.3389/fcimb.2024.1451539

**Published:** 2024-11-28

**Authors:** Zhuo-Yun Tang, Ping Xu, Zhong-Hao Wang, Ting-Ting Wang, Dan Zhou, Ke-Ping Ao, Hua-Feng Song, Xiao-Yun Yin, Dong-Dong Li

**Affiliations:** ^1^ Department of Laboratory Medicine, West China Hospital of Sichuan University, Chengdu, Sichuan, China; ^2^ Department of Clinical Laboratory, The Fifth People’s Hospital of Suzhou, Infectious Disease Hospital Affiliated to Soochow University, Suzhou, Jiangsu, China

**Keywords:** cryptococcal antigen, cryptococcosis, chemiluminescence assay (CLIA), lateral flow assay (LFA), diagnostic performance, treatment monitoring

## Abstract

**Objective:**

This study aimed to assess the efficacy of innovative Chemiluminescence Immunoassay (CLIA) in testing Cryptococcal Antigen (CrAg) across two medical centers, employing the FDA-approved CrAg Lateral Flow Assay (LFA) by IMMY as a reference standard.

**Methods:**

The study encompassed patients diagnosed with cryptococcosis at West China Hospital of Sichuan University (HX) between July 2022 and May 2023, and Suzhou Fifth People’s Hospital (SZ) from September 2020 to September 2023. All specimens underwent simultaneous detection using the LFA (IMMY, Norman, USA) and CLIA (Chuanglan, Suzhou, China).

**Results:**

A total of 628 patients were enrolled, revealing a remarkable 99.20% concordance between LFA and CLIA (623/628, 99.20%). The LFA exhibited a sensitivity of 96.83% (244/252) and specificity of 98.35% (179/182). Among the 42 patients with unaltered CrAg titers, the changes of Signal-to-Cut-Off ratio (ΔS/CO) results exhibited a noteworthy discrepancy, with 71.43% (30/42) demonstrating a decreasing trend in ΔS/CO of at least 10%.

**Conclusions:**

The CLIA method demonstrated commendable specificity and sensitivity, exhibiting a high level of agreement with the FDA-approved LFA method. Additionally, CLIA demonstrated superior utility for treatment monitoring compared to LFA, offering continuous insight into the fluctuation of CrAg concentrations.

## 1 Background

Cryptococcosis constitutes a severe invasive fungal ailment associated with significant morbidity and mortality on a global scale (Benedict et al., 2022; Rajasingham et al., 2022). Cryptococcal antigenemia and meningitis predominantly afflict individuals experiencing immunosuppression due to conditions such as HIV, malignancies, organ transplantation, and similar states (Marr et al., 2020; Liang et al., 2023). While cryptococcal antigenemia and meningitis are prevalent among those with HIV globally (Rajasingham et al., 2017; Bive et al., 2022), an increasing number of non-HIV individuals in China are falling victim to cryptococcosis due to variations in population susceptibility (Chen et al., 2020; Zhao et al., 2021). The recognition of cryptococcosis in seemingly immunocompetent patients is also on the rise (George et al., 2018; Hevey et al., 2019; Li et al., 2021; Pinheiro et al., 2021). Given that early identification and intervention can mitigate morbidity and mortality, comprehending testing trends for cryptococcosis is imperative in estimating its public health impact (Baddley et al., 2021; Benedict et al., 2022; Liang et al., 2023).

Laboratory methodologies employed for cryptococcosis diagnosis typically involve culture, microscopic examination of cerebrospinal fluid (CSF), and the detection of cryptococcal antigen (CrAg) in bodily fluids (Osaigbovo and Bongomin, 2021; Benedict et al., 2022). Among these, CrAg detection stands out as the most expeditious and widely employed diagnostic approach, owing to its elevated sensitivity and specificity (Dubbels et al., 2017; Tugume et al., 2023). Latex agglutination, enzyme immunoassay and lateral flow assay (LFA), represent common techniques for CrAg detection (Kamble et al., 2021; Macrae et al., 2023). CrAg titers serve as prognostic indicators for mortality and can supplement therapeutic assessments (Wang et al., 2020; Tadeo et al., 2021). While LFA are straightforward, rapid, and economical diagnostic methods, determining titers using LFA necessitates an ample supply of strips and reagents, coupled with technical proficiency. The chemiluminescence assay (CLIA) has the advantages of simple operation, low cost, high sensitivity and stable reagents (Li and Guo, 2024). Consequently, CLIA emerges as an appealing alternative, potentially enabling precise management decisions based on titers at a reduced cost (Dubbels et al., 2017; Wang et al., 2020; Macrae et al., 2023; McHale et al., 2023).

The objective of this study was to assess the efficacy of a novel chemiluminescence assay for testing CrAg and CrAg titers in two medical centers, utilizing the FDA-approved CrAg LFA (IMMY) as a benchmark.

## 2 Materials and methods

### 2.1 Cohort population

All patients diagnosed with cryptococcosis at West China Hospital of Sichuan University (HX) between July 2022 and May 2023 and Suzhou Fifth People’s Hospital (SZ) from September 2020 to September 2023 were enrolled in this study. Patient samples with the following conditions were excluded: 1) serum samples with severe hemolysis and lipidemia; 2) CSF samples with fatty turbidity; 3) samples with insufficient amount. Cryptococcal infection was defined as the presence of a positive serum or CSF CrAg test, isolation of Cryptococcus neoformans in culture, or pertinent clinical information. The date of the diagnostic specimen was utilized as the time of diagnosis. Patient demographics, laboratory testing, treatment data, and outcomes were extracted from the medical charts and subsequently analyzed. The negative group comprised individuals with a negative serum or CSF CrAg result determined by LFA (IMMY, Norman, USA). The interference group included cases with conditions such as bacterial encephalitis, viral encephalitis, autoimmune encephalitis, tuberculous encephalitis, rheumatoid factor and systemic lupus erythematosus. All samples used in this study were acquired from remaining samples after clinical testing.

### 2.2 Testing methods

All specimens underwent concurrent assessment using LFA (IMMY, Norman, USA) and CLIA (Chuanglan, Suzhou, China). Qualitative outcomes were categorized as negative or positive, while semiquantitative results were expressed as tiers (IMMY), and quantitative results were presented as numerical values (Chuanglan).

The operation steps were carried out in accordance with the reagent instructions. For LFA, it was a “sandwich” immunochromatographic test strip for the detection of CrAg. A positive test resulted in two lines (the control line and the test line), and a negative test resulted only one line (the control line), and if the band did not appear, the test was invalid. The LFA assay gave semiquantitative results as 1:5, 1:10, 1:20 and so on.

The novel CLIA method used magnetic particle directly to detect the concentration of CrAg in human serum and cerebrospinal fluid based on a two-step immune response sandwiched with bispecific antibodies: 1) The first step was to bind the CrAg to the magnetic particle-coated anti-capsular polysaccharide monoclonal antibody through an immune response. 2) The second step was to add acridine ester-labeled anti-GXM monoclonal antibody to form a complex. 3) After washing, added pre-excitation and excitation solution and its luminescence intensity was positively correlated with the concentration of capsular polysaccharides in serum or cerebrospinal fluid. The range of the kit was 2-160 ng/mL. If the concentration of CrAg < 2ng/mL, it was considered negative; if the sample was above the upper limit of detection, it was reported as greater than that value (>160 ng/mL).

### 2.3 Statistical methods

Categorical variables were scrutinized utilizing Fisher’s exact test and the chi-square test, as deemed appropriate. Continuous variables were analyzed through the t-test and the Mann-Whitney U test if the assumption of normality was violated. P values of <0.05 were considered statistically significant. The analyses were executed using SPSS [V25] (IBM, Armonk, NY, USA) and Origin [V2022] (OriginLab, Northampton, Massachusetts, USA).

## 3 Results

### 3.1 Comparison of the consistency of LFA and CLIA in qualitative results

We conducted a comprehensive review of 901 samples meeting the enrolment criteria for the positive group, negative group, and interference group from September 2020 to September 2023. A total of 273 samples, identified as replicates, were excluded from the analysis. The resultant cohort comprised 628 patients, revealing a noteworthy 99.20% consistency between LFA and CLIA (623/628, 99.20%) (**Table 1**). Among these, 377 samples originated from West China Hospital (HX), while 251 samples were obtained from The Fifth People’s Hospital of Suzhou (SZ). The positive group consisted of 278 samples, the negative group included 330 samples, and 20 samples were designated as interference group. Each group exhibited substantial consistency between the two methods (271/278, 97.48%; 330/330, 100.00%; 20/20, 100.00%). Notably, 5 samples exhibited inconsistent results, and 80.00% (4/5) of the inconsistent samples were serum samples, with merely one originating from CSF (**Table 2**).

**Table 1:** The results of LFA and CLIA in 628 patients.

Test	Results	LFA		Total
		Positive	Negative	
CLIA	Positive	271 (99.63%)	4 (1.12%)	275
	Negative	1 (0.37%)	352 (98.88%)	353
Total		272	356	628

**Table 2:** The baseline results of five inconsistent samples.

Sample	Age	Gender	Clinical diagnosis	Sample type	LFA result	CLIA result (S/CO)
1	48	Male	Cryptococcosis	Serum	Positive(+)	0.076(-)
2	22	Male	Cryptococcal meningitis	CSF	Negative(-)	3.424(+)
3	57	Female	NP^*^	Serum	Positive(+)	0.598(-)
4	40	Male	Cryptococcosis	Serum	Positive(+)	1.236(-)
5	25	Female	Cryptococcosis	Serum	Positive(+)	0.021(-)

^*^NP, not provided.

### 3.2 Specificity and sensitivity of LFA and CLIA

Demographic data were available for 456 patients across the two centers, excluding 22 patients who either missed the clinical diagnosis or could not be diagnosed with cryptococcosis. The cohort from the two centers comprised 155 women and 279 men, with a mean age of 50.35 years (**Table 3**). The LFA demonstrated a sensitivity of 96.83% (244/252) and a specificity of 98.35% (179/182). Similarly, the CLIA exhibited a sensitivity of 97.22% (245/252) and a specificity of 96.70% (176/182). In HX center, a more detailed analysis of 377 samples was carried out with the sensitivity and specificity was 96.96% (126/132) and 98.24% (112/114) for serum samples; the sensitivity and specificity were 100.00% (67/67) and 97.67% (42/43) for CSF samples.

**Table 3:** Baseline characteristics of LFA and CLIA performed by HX and SZ.

	Total	LFA		CLIA	
		Positive	Negative	Positive	Negative
Mean age (year)	50.35	49.21	51.81	49.04	52.09
Gender					
Male	279 (64.29%)	168 (68.85%)	111 (58.42%)	171 (69.80%)	108 (57.14%)
Female	155 (35.71%)	76 (31.15%)	79 (4158%)	74 (30.20%)	81 (42.86%)
Clinical diagnosis					
Cryptococcosis	211 (48.62%)	204 (83.61%)	7 (3.68%)	200 (81.63%)	13 (6.88%)
Cryptococcal meningitis	41 (0.92%)	40 (16.39%)	4 (2.11%)	45 (18.37%)	0 (0)
Non-cryptococcal infections	182 (43.94%)	0 (0)	179 (94.21%)	0 (0)	176 (93.12%)
Total	434	244	190	245	189

### 3.3 Performance evaluation for the same patient surveillance

**Figure 1:**
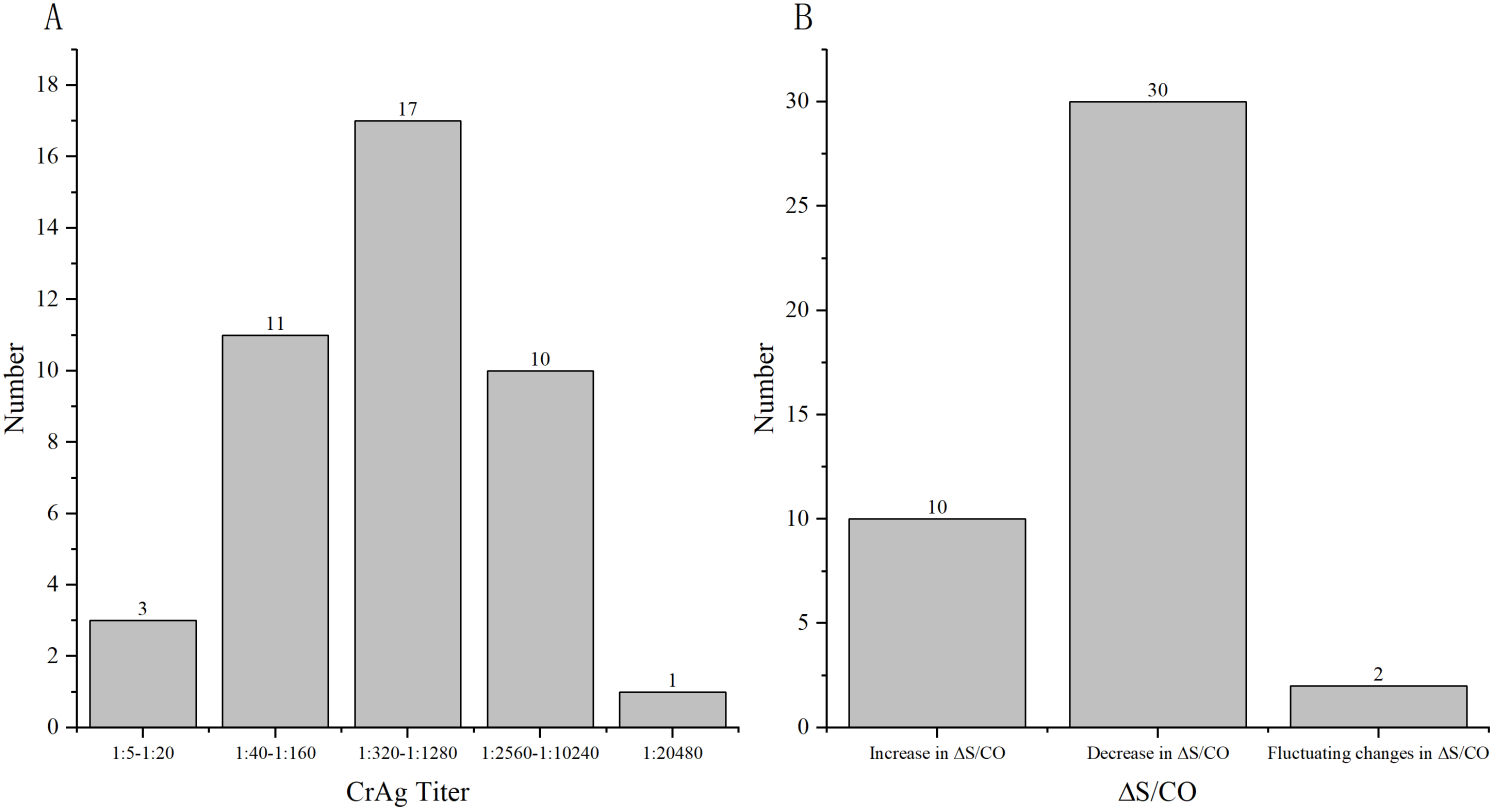
ΔS/CO and LFA results of 42 patients who were available for surveillance. **(A)** The distribution of different CrAg titer of 42 patients by LFA; **(B)** The distribution of different changes in CrAg by CLIA.

**Figure 2:**
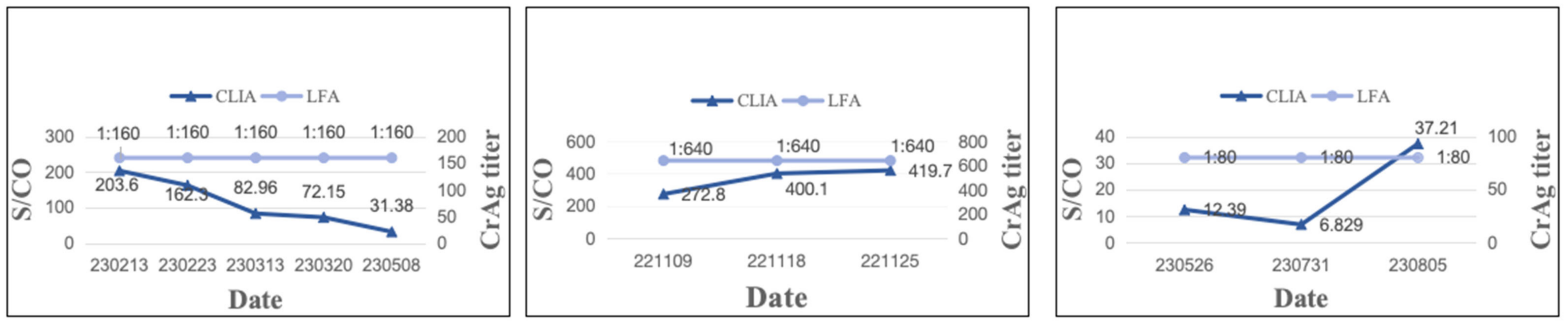
Depiction of three typical patients maintaining the same CrAg titer but exhibiting significantly different S/CO results. Patient 4 had a CrAg titer of 1:160, yet S/CO results decreased. Patient 7 maintained a CrAg titer of 1:640, with increased S/CO results. Patient 16 had a CrAg titer of 1:80, but S/CO displayed fluctuating changes.

A total of 195 samples from 69 patients were available for therapy surveillance, wherein certain patients contributed more than one sample across different time points. All samples underwent both LFA and CLIA testing, revealing 42 patients (60.86%,42/69) maintained the same CrAg titer by LFA but exhibited markedly different CLIA results (ΔS/CO ≥ 10%, ΔS/CO = $\left|\frac{\text{S}/\text{COsecond}-\text{ S}/\text{COfirst}}{\text{S}/\text{COfirst}}\right|\;$ ) (**Figure 1**; **Supplementary Table 1**). The median of ΔS/CO was 0.36 (Interquartile range [IQR], 0.21 to 0.80). Of these patients, 23.81% (10/42) witnessed an increase in ΔS/CO, 71.43% (30/42) experienced a decrease, and 4.76% (2/42) displayed fluctuating changes in CrAg (**Figure 2**).

### 3.4 LFA and CLIA assist in clinical judgment of the timing of drug discontinuation

A compilation of 153 samples with negative fungal cultures of Cryptococcus in CSF was analyzed for LFA, CLIA results, and clinical information on drug discontinuation. Among these samples, only three patients opted for discontinuation of antifungal therapy (**Table 4**). These three patients, all of advanced age, had been orally administered voriconazole for an extended duration of antifungal therapy post-diagnosis. Despite the persistence of CrAg, clinicians chose to discontinue antifungal therapy upon obtaining negative cultures.

**Table 4:** Baseline characteristics of three patients discontinuation of antifungal therapy.

Sample	Age	Gender	Clinical diagnosis	Sample type	CrAg titer (LFA)	S/CO (CLIA)
1	57	Female	Cryptococcal meningitis	Serum	1:10 (+)	3.365 (+)
2	48	Male	Cryptococcosis	Serum	1:2 (+)	0.144 (-)
3	47	Male	Cryptococcal meningitis	Serum	1:80 (+)	8.766 (+)

## 4 Discussion

Cryptococcus capsular polysaccharide antigen stands out as an optimal biomarker for screening cryptococcal infections (Wang et al., 2022; Liang et al., 2023). The LFA currently enjoys global recognition and widespread use for CrAg detection, demonstrating high specificity and sensitivity (Borges et al., 2019; Ware et al., 2023). In contrast to conventional culture, ink staining, and Next-Generation Sequencing (NGS) methods, LFA offers the distinct advantages of speed, accuracy, and cost-effectiveness. However, when utilized for CrAg titer detection, LFA can only provide results in a semi-quantitative format (Skipper et al., 2020; Tenforde et al., 2020). With the increasing adoption of chemiluminescence methodology, the use of the CLIA for CrAg detection addresses the limitations of semi-quantitation, providing results that are more rapid, accurate, and quantitative.

The primary objective of this study was to assess the diagnostic performance of a novel CLIA CrAg assay, using the FDA-approved CrAg LFA test (IMMY) as the reference method. The new CLIA CrAg assay demonstrated perfect consistency with IMMY, showing a 99.20% agreement between LFA and CLIA across the positive group, negative group, and interference group. Both the negative and interference groups exhibited 100.00% consistency, with only 5 out of 278 positive samples displaying inconsistencies. Among these incongruities, 80.00% originated from serum samples, while 20.00% were from CSF. Of the five discordant cases, four were diagnosed with cryptococcal infection, three of which were accurately identified as positive by LFA but incorrectly deemed false-negative by CLIA. One case was falsely negative by LFA but correctly identified as positive by CLIA.

The study evaluated the diagnostic performance of the new CLIA CrAg assay (Chuanglan) in comparison to the LFA CrAg assay (IMMY). Both CLIA and LFA demonstrated excellent test performance, boasting 97.22% sensitivity and 96.70% specificity (CLIA) versus 96.83% sensitivity and 98.35% specificity (LFA). CLIA was more sensitive but less specific. However, the sensitivity and specificity of the IMMY test in this study were marginally lower than in previous studies (Jarvis et al., 2020; Skipper et al., 2020; Kwizera et al., 2021; Noguera et al., 2021; Langner and Yang, 2022), potentially attributed to variations in the testing population (Zhang et al., 2023). Notably, the predominant characteristic of the testing population in China was the prevalence of immunocompetent individuals among cryptococcus-infected patients, in contrast to populations largely comprising immunocompromised individuals such as people living with HIV (PLWH) or organ transplant recipients (Rajasingham et al., 2017; Zhang et al., 2023). Literature reports on non-HIV populations revealed LFA detection performance with 96.00% sensitivity and 96.00% specificity, consistent with our findings (Macrae et al., 2023). This observation is significant as cryptococcal disease, while predominantly affecting PLWH, is increasingly affecting HIV-negative individuals, particularly in regions with advanced healthcare systems.

In this study, 69 patients maintaining the same CrAg titer (IMMY) over an extended period were examined innovatively, revealing that 42 of them experienced a change of more than 10% in S/CO value by CLIA. The median value of ΔS/CO was 36.00%, with 71.43% of patients exhibiting a decreasing trend in CrAg concentration, indicative of the efficacy of antifungal therapy ^[35]^. Conversely, 23.81% and 4.76% of patients showed an increasing and fluctuating trend in CrAg concentration, respectively, suggesting a suboptimal response to antifungal therapy. CrAg concentration serves as an indicator of the rate of cryptococcal clearance, making it a valuable tool for treatment monitoring. The prolonged nature of antifungal therapy renders the LFA method capable of only providing semi-quantitative results, leading to scenarios where the CrAg titer may persist unchanged despite gradual elimination of the pathogen. In such cases, CLIA’s quantitative results offer a superior method for therapeutic monitoring, providing continuous presentation of results and enabling the monitoring of CrAg concentration changes at multiple time points to reflect the efficacy of antifungal therapy.

In this study, we examined 153 patients with negative CSF fungal cultures, subjecting both their serum and CSF to testing with both LFA and CLIA, while reviewing their treatment outcomes. Merely three patients opted to discontinue antifungal therapy upon obtaining negative cultures. The LFA results were 1:2, 1:10, and 1:80, with corresponding CLIA results of 0.144, 3.365, and 8.766, respectively, showing low levels of CrAg. The best time to discontinue the antifungal therapy should be further discussed, and therefore, we proposed a hypothesis and plan to expand the dataset to establish a “gray zone” based on LFA and CLIA results. CrAg values below this “gray zone” might signal antifungal discontinuation, potentially reducing drug side effects and fungal resistance. This prompts the consideration of methodological limitations contributing to the continued detection of CrAg even in the absence of fungal growth.

There were also some shortcomings in this study. Firstly, there were two medical centers, covering only the southwest and eastern parts of China, which was not a good representation of the overall situation in China. Second, there were few data for drug and treatment monitoring, and this part can be further focused on in follow-up studies. In addition, the CLIA assay were not commercially available and it will take time to be used in the clinic.

In conclusion, this study assessed a novel CLIA method for CrAg detection, demonstrating high consistency with the FDA-approved LFA method and exhibiting commendable specificity and sensitivity (both > 96.00%). Owing to methodological disparities, CLIA showcased superior application value compared to LFA for treatment monitoring, offering continuous reflection of CrAg concentration changes. Additionally, we anticipate that in subsequent studies, the establishment of a CrAg “gray zone” for patients with negative cultures could provide supplementary guidance for drug discontinuation decisions.

## Data Availability

The raw data supporting the conclusions of this article will be made available by the authors, without undue reservation.
