# Supplementary material for: Evaluation of cryptococcal antigen testing using a novel chemiluminescence assay in two medical centers of China
Source: Front Cell Infect Microbiol. 2024 Nov 28;14:1451539. doi: 10.3389/fcimb.2024.1451539 (PMC11634829; doi:10.3389/fcimb.2024.1451539)
Supplement: Supplementary file 1 [file Table1.docx]

**Evaluation of Cryptococcal Antigen Testing Using a Novel Chemiluminescence Assays in two medical centers of China**

**Zhuo-Yun Tang^1,#^ , Ping Xu^2,#^ , Zhong-Hao Wang^1^, Ting-ting Wang^1^, Dan Zhou^1^, Ke-Ping Ao^1^, Hua-Feng Song^2^, Xiao-Yun Yin^2^, Dongdong Li^1,*^**

^1^ Department of Laboratory Medicine, West China Hospital of Sichuan University, Chengdu 610041, Sichuan Province, China

^2^ Department of Clinical Laboratory, The Fifth People’s Hospital of Suzhou, Infectious Disease Hospital Affiliated to Soochow University, Suzhou 215000, Jiangsu Province, China

# Zhuo-Yun Tang and Ping Xu contributed equally to this work.

* Corresponding authors: Dongdong Li, MD, [jiangxili1219@163.com](mailto:jiangxili1219@163.com), Tel: 86-28-85422612, Fax: 86-28-85423510

**Table 1. ∆S/CO and LFA results of 42 patients who were available for surveillance.**

| No. patients | Date | Sample type | LFA results (CrAg titer) | CLIA results (S/CO) | ∆S/CO |
| --- | --- | --- | --- | --- | --- |
| 1-1 | 230818 | Serum | 1:2560 | 2365 | 0.13 |
| 1-2 | 230829 | Serum | 1:2560 | 2705 | / |
| 2-1 | 230505 | CSF | 1:2560 | 1245 | 0.21 |
| 2-1 | 230515 | CSF | 1:2560 | 1028 | 0.10 |
| 2-3 | 230609 | CSF | 1:2560 | 932.9 | / |
| 3-1 | 230830 | CSF | 1:2560 | 973.4 | 0.40 |
| 3-2 | 230907 | CSF | 1:2560 | 1606 | 0.20 |
| 3-3 | 230919 | CSF | 1:2560 | 2006 | / |
| 4-1 | 230213 | Serum | 1:160 | 203.6 | 0.25 |
| 4-2 | 230223 | Serum | 1:160 | 162.3 | 0.96 |
| 4-3 | 230313 | Serum | 1:160 | 82.96 | 0.15 |
| 4-4 | 230320 | Serum | 1:160 | 72.15 | 1.30 |
| 4-5 | 230508 | Serum | 1:160 | 31.38 | / |
| 5-1 | 221101 | CSF | 1:5120 | 4805 | 0.32 |
| 5-2 | 221107 | CSF | 1:5120 | 3647 | 0.04 |
| 5-3 | 221116 | CSF | 1:5120 | 3505 | 0.38 |
| 5-4 | 221212 | CSF | 1:5120 | 2534 | 0.24 |
| 5-5 | 230402 | CSF | 1:5120 | 2044 | / |
| 6-1 | 221024 | Serum | 1:80 | 29.68 | 0.15 |
| 6-2 | 221209 | Serum | 1:80 | 25.71 | 0.17 |
| 6-3 | 230315 | Serum | 1:80 | 21.98 | / |
| 7-1 | 221109 | CSF | 1:640 | 272.8 | 0.32 |
| 7-2 | 221118 | CSF | 1:640 | 400.1 | 0.05 |
| 7-3 | 221125 | CSF | 1:640 | 419.7 | / |
| 8-1 | 221207 | Serum | 1:640 | 512.7 | 0.42 |
| 8-2 | 230223 | Serum | 1:640 | 361.9 | / |
| 9-1 | 230420 | CSF | 1:2560 | 1501 | 0.12 |
| 9-2 | 230424 | CSF | 1:2560 | 1336 | / |
| 10-1 | 230309 | Serum | 1:5120 | 7274 | 0.80 |
| 10-2 | 230331 | Serum | 1:5120 | 4046 | 0.78 |
| 10-3 | 230507 | Serum | 1:5120 | 2277 | 0.89 |
| 10-4 | 230528 | Serum | 1:5120 | 1203 | / |
| 11-1 | 221209 | CSF | 1:2560 | 3241 | 0.24 |
| 11-2 | 221216 | CSF | 1:2560 | 2604 | 0.19 |
| 11-3 | 221220 | CSF | 1:2560 | 2196 | 0.31 |
| 11-4 | 221227 | CSF | 1:2560 | 1674 | 0.61 |
| 11-5 | 230214 | CSF | 1:2560 | 938.7 | 0.18 |
| 11-6 | 230321 | CSF | 1:2560 | 793.5 | / |
| 12-1 | 220729 | Serum | 1:640 | 58.68 | 0.48 |
| 12-2 | 220901 | Serum | 1:640 | 112.1 | / |
| 13-1 | 221115 | Serum | 1:640 | 330.9 | 0.63 |
| 13-2 | 230321 | Serum | 1:640 | 901.9 | / |
| 14-1 | 230706 | CSF | 1:80 | 71.99 | 0.121 |
| 14-2 | 230710 | CSF | 1:80 | 64.01 | 4.37 |
| 14-3 | 230807 | CSF | 1:80 | 11.91 | / |
| 15-1 | 220826 | CSF | 1:1280 | 2999 | 0.36 |
| 15-2 | 220901 | CSF | 1:1280 | 2203 | / |
| 16-1 | 230526 | Serum | 1:80 | 12.39 | 0.81 |
| 16-2 | 230731 | Serum | 1:80 | 6.829 | 0.82 |
| 16-3 | 230805 | Serum | 1:80 | 37.21 | / |
| 17-1 | 230613 | Serum | 1:20480 | 16748 | 0.05 |
| 17-2 | 230621 | Serum | 1:20480 | 15915 | 0.04 |
| 17-3 | 230626 | Serum | 1:20480 | 15324 | 0.23 |
| 17-4 | 230701 | Serum | 1:20480 | 12429 | / |
| 18-1 | 221027 | CSF | 1:2560 | 3538 | 0.14 |
| 18-2 | 221102 | CSF | 1:2560 | 3102 | 0.35 |
| 18-3 | 221107 | CSF | 1:2560 | 2305 | 1.20 |
| 18-4 | 221114 | CSF | 1:2560 | 1047 | / |
| 19-1 | 221017 | CSF | 1:640 | 218.1 | 0.36 |
| 19-2 | 221109 | CSF | 1:640 | 342.2 | / |
| 20-1 | 230313 | Serum | 1:40 | 40.25 | 0.05 |
| 20-2 | 230320 | Serum | 1:40 | 38.34 | 1.48 |
| 20-3 | 230327 | Serum | 1:40 | 15.44 | / |
| 21-1 | 230425 | CSF | 1:2560 | 3261 | 0.36 |
| 21-2 | 230516 | CSF | 1:2560 | 2393 | 0.12 |
| 21-3 | 230522 | CSF | 1:2560 | 2706 | / |
| 22-1 | 230613 | Serum | 1:320 | 101.8 | 1.24 |
| 22-2 | 230717 | Serum | 1:320 | 45.54 | / |
| 23-1 | 220719 | Serum | 1:640 | 594.1 | 2.85 |
| 23-2 | 220807 | Serum | 1:640 | 154.3 | / |
| 24-1 | 230720 | Serum | 1:80 | 70.48 | 1.42 |
| 24-2 | 230801 | Serum | 1:80 | 29.18 | 0.04 |
| 24-3 | 230804 | Serum | 1:80 | 28.17 | / |
| 25-1 | 230308 | Serum | 1:640 | 118.7 | 0.12 |
| 25-2 | 230510 | Serum | 1:640 | 106.2 | 0.23 |
| 25-3 | 230626 | Serum | 1:640 | 86.34 | / |
| 26-1 | 230626 | Serum | 1:1280 | 671.2 | 0.34 |
| 26-2 | 230704 | Serum | 1:1280 | 500.5 | 0.11 |
| 26-3 | 230714 | Serum | 1:1280 | 449.6 | 0.65 |
| 26-4 | 230801 | Serum | 1:1280 | 272.8 | / |
| 27-1 | 221213 | Serum | 1:40 | 13.25 | 0.85 |
| 27-2 | 230503 | Serum | 1:40 | 7.158 | / |
| 28-1 | 220822 | Serum | 1:320 | 221.1 | 0.20 |
| 28-2 | 220919 | Serum | 1:320 | 183.9 | 0.78 |
| 28-3 | 221109 | Serum | 1:320 | 103.4 | / |
| 29-1 | 230209 | CSF | 1:320 | 166.2 | 0.03 |
| 29-2 | 230406 | CSF | 1:320 | 171.6 | 4.74 |
| 29-3 | 230615 | CSF | 1:320 | 29.92 | / |
| 30-1 | 221110 | Serum | 1:320 | 283.4 | 0.36 |
| 30-2 | 230308 | Serum | 1:320 | 209.0 | / |
| 31-1 | 230324 | CSF | 1:320 | 364.6 | 0.21 |
| 31-2 | 230331 | CSF | 1:320 | 459.7 | / |
| 32-1 | 221107 | Serum | 1:5 | 4.006 | 0.09 |
| 32-2 | 230324 | Serum | 1:5 | 3.690 | 0.49 |
| 32-3 | 230507 | Serum | 1:5 | 2.471 | 0.09 |
| 32-4 | 230612 | Serum | 1:5 | 2.712 | / |
| 33-1 | 230522 | CSF | 1:2560 | 2242 | 0.36 |
| 33-2 | 230529 | CSF | 1:2560 | 1643 | / |
| 34-1 | 230418 | Serum | 1:20 | 6.343 | 0.295 |
| 34-2 | 230530 | Serum | 1:20 | 4.906 | 1.06 |
| 34-3 | 230709 | Serum | 1:20 | 2.386 | / |
| 35-1 | 221207 | Serum | 1:40 | 18.37 | 1.62 |
| 35-2 | 230507 | Serum | 1:40 | 7.017 | 0.11 |
| 35-3 | 230704 | Serum | 1:40 | 7.855 | / |
| 36-1 | 221019 | CSF | 1:320 | 708.9 | 0.23 |
| 36-2 | 221027 | CSF | 1:320 | 922.8 | 0.13 |
| 36-3 | 221103 | CSF | 1:320 | 1065 | / |
| 37-1 | 220819 | Serum | 1:640 | 1361 | 0.41 |
| 37-2 | 220830 | Serum | 1:640 | 964.5 | 0.29 |
| 37-3 | 220908 | Serum | 1:640 | 748.5 | 0.59 |
| 37-4 | 220915 | Serum | 1:640 | 470.3 | / |
| 38-1 | 220719 | CSF | 1:640 | 10.50 | 3.72 |
| 38-2 | 220721 | CSF | 1:640 | 2.226 | / |
| 39-1 | 221102 | Serum | 1:80 | 30.08 | 1.67 |
| 39-2 | 230310 | Serum | 1:80 | 11.28 | / |
| 40-1 | 221226 | Serum | 1:10 | 5.244 | 0.32 |
| 40-2 | 230308 | Serum | 1:10 | 7.672 | / |
| 41-1 | 230321 | Serum | 1:40 | 13.94 | 0.27 |
| 41-2 | 230329 | Serum | 1:40 | 10.98 | / |
| 42-1 | 230331 | Serum | 1:80 | 15.50 | 0.27 |
| 42-2 | 230507 | Serum | 1:80 | 12.21 | / |
